# Complete chloroplast genome of *Camellia rubituberculata*: a species endemic to Guizhou, China

**DOI:** 10.1080/23802359.2021.1961625

**Published:** 2021-08-09

**Authors:** Yuanyuan Li, Haiyan Liu, Jun Zou

**Affiliations:** a Guizhou Botanical Garden, Guiyang, China; b Guizhou Forestry Bureau, Guiyang, China

**Keywords:** Plastid genome, high-throughput sequencing, phylogenetic analysis, phylogeny

## Abstract

*Camellia rubituberculata* Chang ex Lin et Lu is an endemic woody plant species with ornamental and edible oil value in Guizhou, China. Lack of genetic information seriously hinders its conservation and sustainable utilization. In this study, its complete chloroplast genome was assembled and annotated. Results show that the total length of the genome is 157,137 bp, in which the LSC is 86,782 bp in length, the SSC is 18,279 bp, and the length of the two long repeat regions is 26,038 bp, respectively. A total of 115 genes in the chloroplast genome of C. *rubituberculata* were annotated, including 80 protein-coding genes, 29 tRNA genes and 4 rRNA genes. In addition, there are 19 genes with two copies in the genome. Phylogenetic tree based on 62 homologous coding genes from 24 species chloroplast genomes showed that *C. anlungensis* is the most closely related species of *C. rubituberculata*. These results could enrich the chloroplast genomic information of Theaceae species, and lay a solid foundation for the study of phylogenetic relationships of this family, as well as the conservation and sustainable utilization of *C. rubituberculata*.

*Camellia rubituberculata* Chang ex Lin et Lu is a woody species endemic to Guizhou, China. It is distributed in the subtree layer or shrub layer of the evergreen broad-leaved deciduous mixed forest. It has pink or red flowers with long flowering period, and fruits with nodular protuberance on the surface. *C. rubituberculata*, which retains its original character, is of great significance to the study of the evolution of *Camellia* species. It is also a crucial source of local ornamental plants and edible oils. However, due to the human disturbance caused by its economic value, it has tended to be scarce in recent years and need to be conserved urgently. Lack of genetic information seriously hinders its conservation and sustainable utilization.

The structure and genetic composition of chloroplast genomes in higher plants are relatively conserved with a moderate mutation rate. Chloroplast genome sequences, as the preferred molecular markers for studying plant genetic variation, can provide basic information for the conservation and utilization of valuable plant resources (Niu et al. 2018; Daniell et al. 2021; Yu et al. 2021). Therefore, in this study, we characterize the chloroplast genome of *C. rubituberculata* in order to provide basic information for the conservation of the genetic resources.

Leaf samples were collected from Guizhou Province, China (E105.26°, N25.61°, H 1350 m). A specimen was deposited at the herbarium of Guizhou Botanical Garden (Yuanyuan Li, gzszwylyy@163.com) under the voucher number (GZY20210176). Whole genomic DNA was isolated using Plant Genomic DNA Extraction Kit (Tiangen Beijing China). After the DNA sample was qualified by Beijing Novogene Bio-technology Co., Ltd., Illumina double-end sequencing technology was used for library construction, and the construction type was 400 bp DNA fragment. Raw data were trimmed and used to de novo assemble of contigs of the chloroplast genome of *C. rubituberculata* by using CLC workbench v10 (CLC Bio, Denmark). The assembled contigs with length above 10,000 bp were blasted against database of NCBI, the hit complete chloroplast genome of relative species with the highest homology was then selected as reference genome to assemble of the complete chloroplast genome of C. *rubituberculata* by using software MITObim v1.7 (Hahn et al. 2013). Genome annotation was performed by comparing the chloroplast genome with that of selected relative species from GenBank, and by online software GeSeq (Tillich et al. 2017), which was then manually corrected after annotation. The homologous coding gene sequences were extracted from chloroplast genomes of C. *rubituberculata*, 22 related *Camellia* species, and *Populus wilsonii*, and were aligned, and concatenated in Geneious v11, then imported to MEGA X for phylogenetic tree construction based on maximum likelihood (Kumar et al. 2018).

A total of 410,528 reads were used to assemble of the chloroplast genome of *C. rubituberculata* with a mean coverage of 392.4×. The genome is a closed circular molecule that is relatively conserved and has a typical tetrad structure. The total length of the genome is 157,137 bp, and the GC content is 37.3%, in which the large single-copy region is 86,782 bp in length, and the GC content is 35.3% GC, whereas the small single-copy region is 18,279 bp, and the GC content is 30.6%. The length of the two reverse repeat regions is 26,038 bp and the GC content is 43%, respectively.

A total of 115 genes in the chloroplast genome of C. *rubituberculata* were annotated successfully, including 80 protein-coding genes, 29 tRNA genes and 4 rRNA genes and two pseudogenes (rps19 and ycf1). Among which, ycf3 and clpP genes contain two introns, 17 genes contained one intron, including 6 tRNA genes (*trnV-UAG*, *trnL-UAA*, *trnk-UUU*, *trnI-GAU*, *trnG-UCC*, and *trnA-UGC*) and 11 protein-coding genes (*rps16*, *rpoC1*, *rpl16*, *rpl2*, *petB*, *petD*, *ndhA*, *ndhB*, *atpF*, *ycf15*, *ycf4*). In addition, there are 19 genes with two copies in the genome, including four rRNA genes (*rrn4.5*, *rrn 5*, *rrn 16, rrn 23*), seven tRNA-genes (*trnA-UGC*, *trnH-CAU*, *trnI-GAU*, *trnL-CAA*, *trnN-GUU*, *trnR-ACG*, *trnV-GAC*), and eight coding genes (*ndhB*, *rpl2*, *rpl23*, *rps7*, *rps12*, *rps19*, *ycf2*, *ycf15*).

Phylogenetic tree was constructed based on 62 homologous coding gene of chloroplast genomes from the 24 species, including *accD*, *atpA*, *atpB*, *atpE*, *atpF*, *atpH*, *atpI*, *ccsA*, *cemA*, *clpP*, *matK*, *ndhC*, *ndhD*, *ndhE*, *ndhF*, *ndhG*, *ndhH*, *ndhI*, *ndhJ*, *ndhK*, *petA*, *petB*, *petD*, *petG*, *petL*, *petN*, *psaA*, *psaB*, *psaC*, *psaI*, *psbA*, *psbB*, *psbC*, *psbD*, *psbE*, *psbI*, *psbK*, *psbL*, *psbM*, *psbN*, *psbT*, *rbcL*, *rpl14*, *rpl16*, *rpl20*, *rpl22*, *rpl33*, *rpl36*, *rpoA*, *rpoB*, *rpoC1*, *rpoC2*, *rps2*, *rps3*, *rps4*, *rps8*, *rps11*, *rps14*, *rps15*, *rps18*, *ycf3*, and *ycf4* (Figure 1). The results showed that all *Camellia* species clustered into a cluster, in which, *C. rubituberculata* and *C. anlungensis* (Zhu et al. 2020) clustered into the same sect. *Tuberculatae* with a bootstrap support value of 99, indicating that *C. rubituberculata* has the closest relationship with *C. anlungensis*. Moreover, this sect. is a sister group with sect. *Thea* and *Archecamellia*. However, the phylogenetic relationship of *Camellia* species in this study is obviously inconsistent with the with the traditional taxonomy (Min and Bartholomew 2007), which has also been reported in previous studies (Kim et al. 2017; Wang et al. 2017), indicating that the section-level taxonomy of *Camellia* needs to be revised urgently.

Results of this study could enrich the chloroplast genomic information of Theaceae species, and lay a solid foundation for the study of phylogenetic relationships of this family, as well as the conservation and sustainable utilization of C. *rubituberculata*.

## Data Availability

The complete chloroplast genome constructed in study is openly available in the Genome Warehouse Database of National Genomics Data Center (NGDC), China National Center for Bioinformation/Beijing Institute of Genomics, Chinese Academy of Sciences, under accession number GWHBAVO00000000 at https://bigd.big.ac.cn/gwh. The sequence data are openly available in the Genome Sequence Archive in NGDC of China at https://bigd.big.ac.cn/gsa/browse/CRA004142 under accession number CRA004142. The genome sequence has also been deposited in GenBank of NCBI under the accession no. MZ424202. The raw reads deposited in NCBI are under BioProject PRJNA740097, Bio-Sample SAMN19819838, and SRA SRX11202720, respectively. Phylogenetic tree based on 62 homologous coding gene sequences of 24 completed chloroplast genomes. *Represents the the chloroplast genome in this study.
